# Experimentally comparing the attractiveness of domestic lights to insects: Do LEDs attract fewer insects than conventional light types?

**DOI:** 10.1002/ece3.2527

**Published:** 2016-10-13

**Authors:** Andrew Wakefield, Moth Broyles, Emma L. Stone, Gareth Jones, Stephen Harris

**Affiliations:** ^1^ School of Biological Sciences, Life Sciences BuildingUniversity of BristolBristolUK

**Keywords:** broad‐spectrum lighting, Ceratopogonidae, color temperature, compact fluorescent lights, *Culicoides*, disease, filament, light‐emitting diode, vector

## Abstract

LED lighting is predicted to constitute 70% of the outdoor and residential lighting markets by 2020. While the use of LEDs promotes energy and cost savings relative to traditional lighting technologies, little is known about the effects these broad‐spectrum “white” lights will have on wildlife, human health, animal welfare, and disease transmission. We conducted field experiments to compare the relative attractiveness of four commercially available “domestic” lights, one traditional (tungsten filament) and three modern (compact fluorescent, “cool‐white” LED and “warm‐white” LED), to aerial insects, particularly Diptera. We found that LEDs attracted significantly fewer insects than other light sources, but found no significant difference in attraction between the “cool‐” and “warm‐white” LEDs. Fewer flies were attracted to LEDs than alternate light sources, including fewer *Culicoides* midges (Diptera: Ceratopogonidae). Use of LEDs has the potential to mitigate disturbances to wildlife and occurrences of insect‐borne diseases relative to competing lighting technologies. However, we discuss the risks associated with broad‐spectrum lighting and net increases in lighting resulting from reduced costs of LED technology.

## 1 Introduction

Insects exhibit phototactic behavior in response to numerous light properties including specific spectral emissions (Nabli, Bailey, & Necibi, 1999; Somers‐Yeates, Hodgson, McGregor, Spalding, & Ffrench‐Constant, 2013; Van Langevelde, Ettema, Donners, WallisDeVries, & Groenendijk, 2011), polarization (Horváth, Kriska, Malik, & Robertson, 2009), and flicker (Inger, Bennie, Davies, & Gaston, 2014). Of these, changes in lighting spectra have received most attention. Different wavelengths of light vary in their attractiveness to insect orders (Van Grunsven et al., 2014) and families of moths (Somers‐Yeates et al., 2013), with shorter wavelengths including ultraviolet (UV) (<380 nm) being more attractive overall for macro‐moth species than longer wavelengths (Van Langevelde et al., 2011). However, initial findings from a long‐term population‐level study show no difference in moth family attraction to a range of LED spectral emissions (Spoelstra et al., 2015). The presence of UV light, even in small quantities, has a disproportionate attraction for insects (Barghini & de Medeiros, 2012). Experiments using UV filters found a decrease in insect attraction to street lights (Barghini & de Medeiros, 2012; Eisenbeis, 2006) and reduced numbers of “pest” species entering greenhouses (Costa, Robb, & Wilen, 2002; Nguyen, Borgemeister, Max, & Poehling, 2009).

Typically LEDs do not emit UV light and are more energy‐efficient than traditional technologies. Electrical energy is converted almost entirely into electromagnetic (EM) radiation within the range of the visible light spectrum (400–700 nm) and therefore not wasted on producing wavelengths of light invisible to the human eye, that is, UV and infrared (IR). Thus, LEDs are less attractive to many insects than lights which emit UV light (Eisenbeis & Eick, 2011; Van Grunsven et al., 2014). Increased demand for LEDs has led to improvements in energy efficiency and versatility. Consumers can now choose from a range of different correlated color temperatures (CCTs). CCT is a measure of the perceived warmth of a light, measured in Kelvin, with higher values indicating “cooler” CCTs. Typically three categories exist: “cool‐white” (4,000–5,000 K), “neutral‐white” (~4,000 K), and “warm‐white” (2,700–3,000 K) (Integral LED 2015). CCT is an inadequate metric to measure the emissions of a light, although it is commonly used by lighting engineers and retailers to describe lighting products. While an early study found that “cool‐white” LEDs attract more insects than “neutral/warm‐white” LEDs (Eisenbeis & Eick, 2011), a more recent study concluded that insects are equally attracted to a range of “white” LEDs which differ subtly in their spectral output (Pawson & Bader, 2014). Longcore et al. (2015) reported that arthropod attraction to “white” LEDs can be minimized by customizing the emission spectra of LEDs. As LEDs are predicted to occupy almost 70% of the general lighting market and over 70% of the outdoor and residential lighting markets by 2020 (Baumgartner et al., 2012), it is important to determine how different “white” LEDs may affect wildlife and ecosystems (Gaston, Visser, & Holker, 2015).

Worldwide, a range of insect taxa, including several families of Diptera, damage crop production (Dosdall et al., 2012; Oerke, 2006; Walsh, Prendergast, Sheridan, & Murphy, 2013). Flies (e.g., *Culicoides* spp., Ceratopogonidae) also have an impact on livestock survival and welfare (Du Toit, 1944; Mehlhorn et al., 2007). With the predicted increase in the world's human population (United Nations Department of Economic and Social Affairs Population Division, 2013), the ability to minimize losses in food production sustainably is an important issue and one in which light‐emitting diodes (LEDs) could feature. Electrophysiological and behavioral experiments are in progress to determine how LEDs can be used in “integrated pest management” techniques both to attract and repel agricultural pests and to predict outbreaks (Shimoda & Honda, 2013). Insects that are disease vectors also pose a threat to human health when attracted to artificial lights (Barghini & de Medeiros, 2010). Currently many nations in tropical and subtropical areas are experiencing rapid economic growth. This will lead to social development and an associated increase in the amount of outdoor lighting. Combined with an increase in the percentage of people living in urban (and so artificially lit) areas (López Moreno, Oyeyinka, & Mboup, 2010), the potential for artificial light at night to facilitate the spread and occurrence of insect‐borne pathogens needs urgent attention. There is clearly a need to develop lighting which limits disturbances to wildlife, restricts losses from agricultural systems, and minimizes human health consequences.

We compared the attractiveness of traditional (tungsten filament) and modern (compact fluorescent and LEDs) “domestic” lights typically used outdoors, for example, on porches and external walls, to insects. We tested two main hypotheses: (i) “Domestic” LEDs attract fewer insects than competing modern and traditional light sources, and (ii) insect attraction differs between “cool‐white” and “warm‐white” LED lighting.

## 2 Materials and methods

### 2.1 Study sites

**Figure 1:**
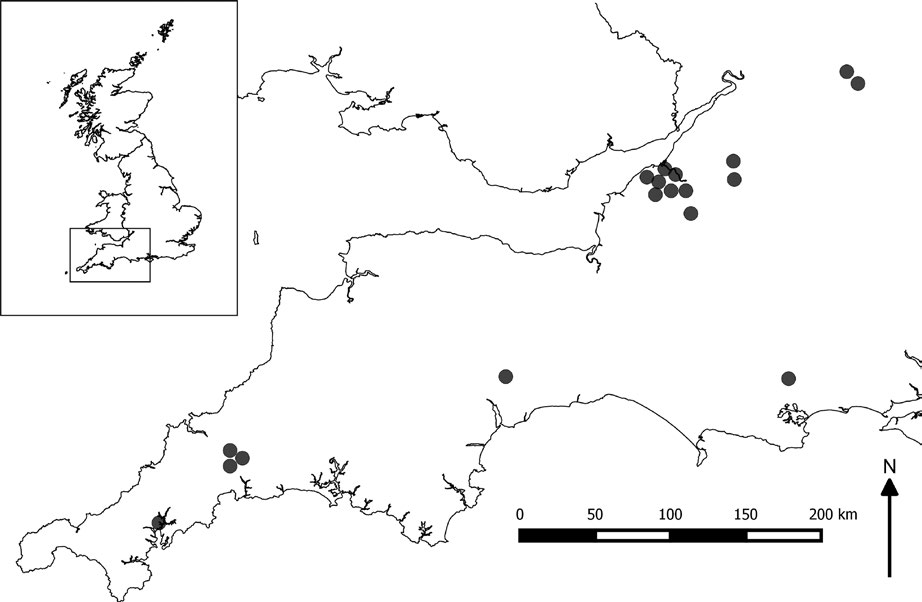
Map of southwest England, UK, showing the locations of the 18 sites where field experiments were conducted between 15 July and 10 September 2014

Experiments were conducted at 18 sites across southern England between 15 July and 10 September 2014. Sites were located on average 101 km apart (range = 0.5–288 km) and experiments took place for one night only at each site. This design aimed to (a) maximize the diversity of insects caught, (b) reduce the impact of the experiment on local insect populations, and (c) generate a clearer picture of how outdoor domestic lights may affect insects nationally (Figure 1). Experiments were conducted in open grassland (meadow or grazed pasture measuring at least 120 × 120 m) which served as largely homogenous habitats in which insect attraction could be attributed to light type with confidence. The choice of grassland habitat also reflects the lights’ application, as they are suitable for use in domestic outdoor environments, such as on porches and external walls illuminating garden lawns. All sites were >100 m from existing artificial lighting and therefore relatively unaffected by other lights. Our replicated experimental design (presenting all light sources on the same night) controlled for factors such as moon phase and temperature that are known to affect insect activity and attractiveness (Bishop et al., 2000).

### 2.2 Lighting equipment and setup

**Figure 2:**
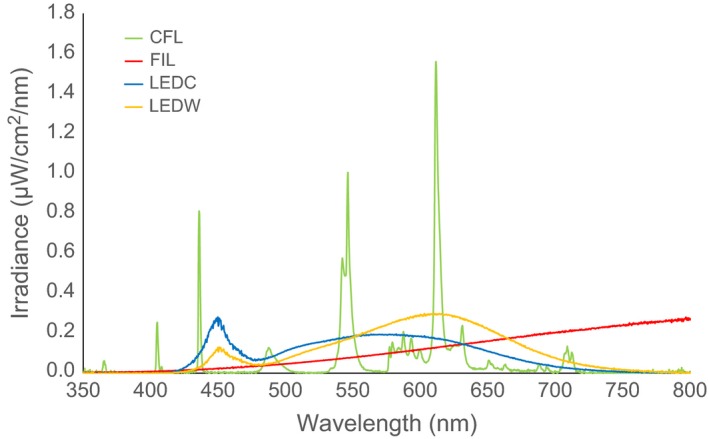
Spectral distribution of the four light types used in this study: compact fluorescent (CFL), tungsten filament (FIL), light‐emitting diode “cool‐white” (LEDC) and light‐emitting diode “warm‐white” (LEDW). Two identical bulbs per light type were used in this study; for clarity, only emissions from one of each are represented graphically

Four different domestic lights were tested—compact fluorescent (CFL), tungsten filament (FIL), “cool‐white” LED (LEDC), and “warm‐white” LED (LEDW) (Table 1). Lights were selected with advice from lighting engineers at integral LED (Iron Bridge Business Park, London, UK), ensuring that the two LED lights were closely matched in every property except CCT (spectral distribution) and that the CFL and FIL bulbs were closely matched to the LEDW bulb for size, shape, lumen output, and CCT. Spectral measurements (Figure 2) were taken in a darkened room using a cosine corrector at the end of a 200‐μm‐diameter ultraviolet–visible fiber optic cable connected to a spectrometer (USB2000, Ocean Optics, Dunedin, Florida, USA) controlled by a PC running SpectraSuite (Version 6, Ocean Optics). The end of the fiber with the cosine corrector was positioned 100 cm away, directed toward the side of each light.

**Table 1:** Product information for the four lights used in this experiment

Lamp	Abbreviation	Luminance (lm)	Wattage (W)	CCT	Model name	Manufacturer
Compact fluorescent	CFL	1,100	20	2,700 K	Compact Fluorescent GLS Bulb	British Electric Lamps Ltd.
Tungsten filament	FIL	970	100	2,700 K	Professional GLS	Crompton Lamps
LED (cool‐white)	LEDC	1,130	13	5,000 K	Classic Globe	Integral LED
LED (warm‐white)	LEDW	1,060	13	2,700 K	Classic Globe	Integral LED

Each light was suspended 1.3 m above the ground using two lengths of metal doweling clamped at right angles to one another (Fig. S1). Each light holder was screwed to a block of wood which was attached to the horizontal length of doweling using waterproof tape, such that each light was always 20 cm away from the vertical doweling for consistency. Lights were powered using 12 V, 22 Ah lithium polymer battery packs (Deben Group Industries, Woodbridge, Suffolk, UK) connected via a 300‐W current inverter (Toolstation, Bridgwater, UK). Low wattage CFL and LED lights required a single battery pack per light, whereas the 100‐W FIL lights required two battery packs connected in parallel to power the light for 4 hr continuously. The inverter was connected to the light socket with arctic flex cabling (1.5 mm 3‐core) suitable for exterior use. The battery packs and current inverters were housed in a weatherproof plastic box at the base of each light stand. Insects were collected in custom‐made insect traps consisting of a 16‐cm‐wide funnel and a 400‐ml plastic beaker half‐filled with water and a couple of drops of detergent to act as a surfactant. The funnel was attached to the light holder using fishing wire and the beaker attached to the funnel using waterproof tape.

Lights were deployed in a square formation, spaced 40 m apart to ensure they were tested in the same habitat and microclimatic conditions but not so close such that insects attracted to one light may have been caught at another. This distance between traps was more conservative than that used in similar studies (e.g., Longcore et al., 2015). Positioning of lights around the square was randomized between sites to control for any orientation biases. Eight individual lights were used (two of each type), although only one of each light type was used per night. Rigorous quality control standards by the lighting manufacturers ensured that the two units for each light had fundamentally identical spectral emissions; they were considered to be identical for the purposes of statistical analyses (see below).

All of the lights were turned on within 10 min of sunset. The FIL light was turned off after 4 hr due its high energy consumption, when insect collection beakers were removed from all four of the light traps. Empty collection beakers were then immediately installed on the remaining three lights (CFL, LEDC, and LEDW) which remained on until sunrise (±2 min). This allowed for comparison between all lights across the same timescale each night and to separate insects into “evening” and “morning” samples. The latter is important for investigating the proportion of insects that arrive at lights within 4 hr after sunset, when many lights will be operated in domestic and commercial settings. Average sampling duration during the “evening” trapping period was 240 min (range = 237–243, *n* = 17 sites), during the “morning” was 305 min (range = 227–420, *n* = 12 sites), and for the whole night was 553 min (range = 487–660, *n* = 11 sites). Insect sampling was undertaken on nights with a favorable weather forecast, that is, no rain and wind speed <12 mph.

### 2.3 Insect collection and identification

Captured insects were frozen for at least 48 hr, pinned or immersed in ethanol (>70%), and identified to family level (Barnard & Ross, 2012; Chinery, 1993; Elliot & Humpesch, 1983; Luff, 2007; New, 2006; Oosterbroek, 2006; Plant, 1997; Sterling, Parsons, & Lewington, 2012; Unwin, 1981, 1984, 2001; Waring, Townsend, Tunmore, & Lewington, 2009; Watson & Dallwitz, 2003). Biting midges in the family Ceratopogonidae were identified to genus level (Boorman & Rowland, 1988).

### 2.4 Statistical analyses

Equipment failures during one “evening” and six “morning” trapping sessions meant that analyses of “evening” catches included data from 17 sites, analyses of “morning” catches 12 sites, and analyses of “all‐night” catches (pooled “evening” and “morning” data) 11 sites. “Morning” and “all‐night” data were used only for comparison of CFL and LED lights, because the FIL light was only operated during “evening” trapping periods.

Insect catch data from “evening” light traps were analyzed by fitting generalized linear mixed models (GLMMs) with Poisson error structures using the package lme4 (Bates, Maechler, Bolker, & Walker, 2014) in R (version 3.0.1. 2014). For each GLMM, goodness of fit was tested using the package aods3 (Lesnoff & Lancelot, 2013) to ensure the data were not overdispersed. Negative binomial GLMMs were constructed where overdispersion (residual deviance > degrees of freedom) was detected (Crawley, 2008). Each model was then compared to a subsequent model lacking the fixed effect term “light” (four levels: CFL, FIL, LEDC, and LEDW) to examine both the change in deviance between the corresponding models as well as the difference in Akaike information criterion (AIC) values (Zuur, Ieno, Walker, Saveliev, & Smith, 2009). Pairwise comparisons between the different light types were then conducted using Tukey contrasts via the R package multcomp (Hothorn, Bretz, & Westfall, 2008).

To examine whether domestic LEDs attract fewer insects than competing modern and traditional light sources, GLMMs with Poisson error distributions were constructed to analyze “evening” data. Response variables included total number of insects, total Diptera, total Lepidoptera, and total Ceratopogonidae. For each model, “light” was included as a fixed effect and “light position” (the randomized location of each light around each square formation) nested within “site” was included as a random effect. Negative binomial GLMMs with the same fixed and random terms were also constructed to analyze “all‐night” data. It was not feasible to analyze other insect orders or other disease vector families (e.g., Culicidae and Muscidae) due to relatively small catches.

To compare the difference in insect attraction between “cool‐white” and “warm‐white” LED lighting, data were analyzed using outputs from pairwise comparisons of the Poisson and negative binomial GLMMs constructed to determine whether “domestic” LEDs attract fewer insects than competing modern and traditional light sources. “Evening” catches of the most abundant families of Diptera and Lepidoptera were also analyzed using GLMMs with Poisson error distributions.

## 3 Results

Of the 4,086 invertebrates we caught, 4,046 were insects. These included 3,118 Diptera, 698 Lepidoptera, 89 Hymenoptera, 65 Hemiptera, 55 Coleoptera, 11 Trichoptera, four Psocoptera, two Dermaptera, two Ephemeroptera, and two Neuroptera. The 28 mites (Acari) and 12 springtails (11 Symphypleona and one Entomobryomorpha) were not included in the analyses. Total insect catch data from all 18 sites were analyzed for spatial autocorrelation using the package ape (Paradis et al., 2016) in R. Based on the results of a Moran's I test, we accepted the null hypothesis of zero spatial autocorrelation among total insect captures (*p* = .221). Therefore, we are confident that insect captures across sites were independent of one another. On average, the CFL, LEDC, and LEDW caught 48, 19, and 25 insects per “evening” (*n* = 17) and 23, 11, and 10 insects per “morning” (*n* = 12) trapping sessions. At the 11 sites where CFL, LEDC, and LEDW lights were successfully deployed throughout the whole night, a mean of 22, 12, and 14 were caught during “evening” and 25, 12, and 10 during “morning” trapping sessions, respectively, for CFL, LEDC, and LEDW lights.

- Do “domestic” LEDs attract fewer insects than competing modern and traditional light sources?

**Figure 3:**
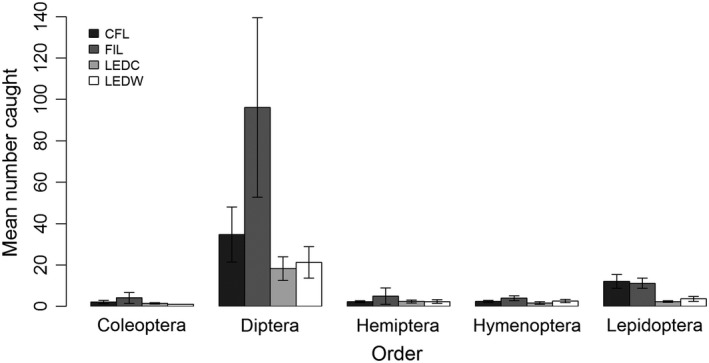
Average insect trap catch per light for the five most abundant insect orders caught in the “evening” sampling periods commencing at sunset (17 sites). Lights were compact fluorescent (CFL), tungsten filament (FIL), light‐emitting diode “cool‐white” (LEDC), and light‐emitting diode “warm‐white” (LEDW). Error bars indicate SE

**Figure 4:**
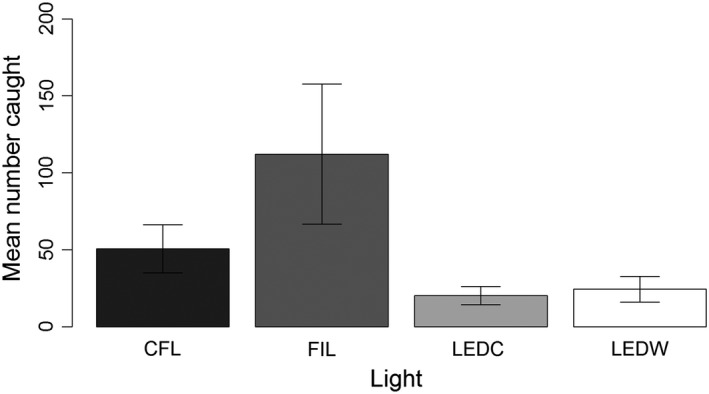
Average insect trap catch in the “evening” sampling periods commencing at sunset (17 sites). Lights were compact fluorescent (CFL), tungsten filament (FIL), light‐emitting diode “cool‐white” (LEDC), and light‐emitting diode “warm‐white” (LEDW). Error bars indicate SE

A total of 3,350 insects were caught during “evening” sampling periods: 2,709 Diptera, 452 Lepidoptera, 65 Hymenoptera, 55 Hemiptera, 51 Coleoptera, nine Trichoptera, three Psocoptera, two Dermaptera, two Ephemeroptera, and two Neuroptera (Table S1). Captures of the five most abundant orders are shown in Figure 3. Thirty‐five families of Diptera were recorded across all lights, with 24 families at CFL, 24 at FIL, 19 at LEDC, and 23 at LEDW. Of the 2,709 flies caught during evening sampling periods, 41% were Lonchopteridae, 15% Ceratopogonidae, 10% Cecidomyiidae, 6% Chironomidae, and 5% Scathophagidae; the remaining 23% belonged to the families shown in Fig. S2. The CFL, FIL, LEDC, and LEDW lights caught 24%, 54%, 10%, and 12% of the total catch, respectively (Figure 4). Light type had a significant effect on total insect attraction based on both Δdeviance and AIC comparison (Table 2a). Pairwise comparison results indicate that significantly fewer insects were attracted to each of the LED lights relative to CFL and FIL lights and that the difference between CFL and FIL lights was approaching significance (Table 3a). The number of insects caught at one site was unusually large, especially for the FIL lamp (Fig. S3). Pairwise comparisons from a GLMM excluding this site showed a more conservative nonsignificant difference in insect attraction between CFL and FIL lights (Table S2).

**Table 2:** Results of model comparisons, in each case between two models with identical random effects but which differed in the inclusion of the single fixed term “light”

	*df*	AIC	Deviance	Chi sq	*df*	*p*
a)						
Light term absent	3	593.79	587.79			
Light term present	6	564.94	552.94	34.847	3	<.001
b)						
Light term absent	3	546.68	540.68			
Light term present	6	533.70	521.70	18.983	3	<.001
c)						
Light term absent	3	396.10	390.10			
Light term present	6	339.44	327.44	62.661	3	<.001

a) Response variable = total insects caught.

b) Response variable = total Diptera caught.

c) Response variable = total Lepidoptera caught.

**Table 3:** Results of multiple comparison tests applied to GLMMs for “evening” insect catches (17 sites); (a) total insects; (b) Diptera; and (c) Lepidoptera. In all models, “light position” nested within “site” was included as a random effect and “light” as the only fixed effect term. Lights were compact fluorescent (CFL), filament (FIL), “cool‐white” light‐emitting diode (LEDC), and “warm‐white” light‐emitting diode (LEDW). * indicates a significant (*p *< .05) difference

	Estimate	SE	Z value	*p*
a)				
FIL–CFL	0.473	0.191	2.485	.063
LEDC–CFL	−0.786	0.201	−3.919	<.001*
LEDW–CFL	−0.660	0.200	−3.305	.005*
LEDC–FIL	−1.260	0.199	−6.348	<.001*
LEDW–FIL	−1.133	0.197	−5.744	<.001*
LEDW–LEDC	0.127	0.207	0.612	.928
b)				
FIL–CFL	0.619	0.238	2.605	.045*
LEDC–CFL	−0.461	0.246	−1.872	.240
LEDW–CFL	−0.371	0.246	−1.509	.432
LEDC–FIL	−1.080	0.243	−4.450	<.001*
LEDW–FIL	−0.991	0.242	−4.090	<.001*
LEDW–LEDC	0.090	0.250	0.358	.984
c)				
FIL–CFL	−0.023	0.142	−0.158	.999
LEDC–CFL	−1.900	0.225	−8.463	<.001*
LEDW–CFL	−1.333	0.186	−7.169	<.001*
LEDC–FIL	−1.877	0.224	−8.368	<.001*
LEDW–FIL	−1.311	0.189	−6.947	<.001*
LEDW–LEDC	0.566	0.256	2.217	.114

Removing the fixed term “light” for “all‐night” data comparisons with negative binomial GLMMs (*n* = 11 sites) resulted in a significantly poorer fit measured by both the chi‐square test for Δdeviance (χ^2^ = 13.902, *df* = 2, *p *< .001) and the AIC value (501.5 with vs. 511.4 without the fixed term). Pairwise comparisons showed a significant difference between the number of insects caught at the CFL and each of the LED light traps (Table S3).

Light type had a significant effect on Diptera attraction based on both Δdeviance and AIC comparison (Table 2b). The FIL light attracted significantly more Diptera than any of the other lights, but there was no significant difference in Diptera attraction between the CFL light and either of the LEDs (Table 3b).

Light type also had a significant effect on Lepidoptera attraction based on both Δdeviance and AIC comparison (Table 2c). Both LEDC and LEDW attracted significantly fewer Lepidoptera than the CFL and FIL lights; there was no significant difference between the number of Lepidoptera caught at CFL and FIL lights (Table 3c).

In total, 398 flies belonging to the family Ceratopogonidae were caught. Other families of Diptera known to be vectors of disease were caught in much smaller numbers, for example, Culicidae (2) and Muscidae (14). The CFL, FIL, LEDC, and LEDW lights caught 15%, 80%, 2%, and 3% of the total Ceratopogonidae catch, respectively. Removing the fixed term “light” resulted in a significantly poorer fitting model when assessed by both Δdeviance (χ^2^ = 42.807, *df* = 3, *p *< .001) and the AIC value (169.35 with vs. 206.16 without the fixed term). Significantly fewer Ceratopogonidae were caught at LEDs than at CFL and FIL lights, and significantly more Ceratopogonidae were caught at FIL lights than CFL lights (Table 4). All Ceratopogonidae caught were identified as *Culicoides* spp.

**Table 4:** Results of multiple comparison tests applied to a Poisson‐distributed GLMM for “evening” Ceratopogonidae catches (17 sites). “Light position” nested within “site” was included as a random effect term and “light” as the only fixed effect term. Lights were compact fluorescent (CFL), filament (FIL), cool‐white light‐emitting diode (LEDC), and warm‐white light‐emitting diode (LEDW). * indicates a significant (*p *< .05) difference

	Estimate	SE	Z value	*p*
FIL–CFL	1.7253	0.1438	12.000	<.001*
LEDC–CFL	−2.0972	0.4005	−5.236	<.001*
LEDW–CFL	−1.4781	0.3074	−4.809	<.001*
LEDC–FIL	−3.8224	0.3821	−10.004	<.001*
LEDW–FIL	−3.2034	0.2829	−11.322	<.001*
LEDW–LEDC	0.6190	0.4688	1.320	.520

- Does insect attraction differ between “cool‐white” and “warm‐white” LED lighting?

Pairwise comparisons of the Poisson‐distributed GLMMs constructed for (i) showed no significant difference in total insect, Diptera, or Lepidoptera attraction between LEDC and LEDW lamps during “evening” trapping periods (Table 3). Similarly, pairwise comparisons of the negative binomial GLMM (“all‐night” data) show no significant difference in insect attraction between LEDC and LEDW (Table S3).

There was no significant difference between the “evening” catches of LEDC and LEDW for any of the three most abundant dipteran families: Cecidomyiidae (*p* = .054), Ceratopogonidae (*p* = .520), and Lonchopteridae (*p* = .996). For the two most abundant lepidopteran families caught, we found that significantly more Crambidae were attracted to LEDW than LEDC (*p* = .01) but no significant difference in Noctuidae captures (*p* = .915).

## 4 Discussion

LEDs (both “cool‐white” and “warm‐white”) caught approximately half as many insects as modern CFL lighting of similar specification and approximately four times fewer insects than traditional FIL lighting. LEDs caught significantly fewer Lepidoptera than CFL and FIL lights and significantly fewer Diptera than FIL lights. Our results provide evidence that LEDs are less attractive to *Culicoides* biting flies (Diptera: Ceratopogonidae) than the other lighting technologies we studied.

Typically, insect vision is either di‐ or trichromatic, with peak sensitivities shifted toward the UV end of the EM spectrum (<380 nm) (Land & Nilsson, 2012). The emission of UV radiation is therefore likely to be the cause of greater insect attraction to CFL lights (Poiani, Dietrich, Barroso, & Costa‐Leonardo, 2015), but not for UV‐absent FIL bulbs. Without detailed information on the visual sensitivities of each species, it is unclear why insect attraction to the FIL light was significantly greater than to LEDs. While trichromatic UV–blue–green vision is common among insects, there are numerous exceptions (Briscoe & Chittka, 2001). Some insects (such as Lepidoptera and Odonata) possess four or five visual pigments (Land & Nilsson, 2012), while *Drosophila* possess seven. The red flour beetle, *Tribolium castaneum*, has dichromatic vision, but is unusual because it has visual pigments at each end of the visible light spectrum—a UV opsin and long‐wavelength opsin (Tribolium Genome Sequencing Consortium, 2008). Some flies are also attracted to red and green light (Green, 1985). Therefore, slight differences in insect visual capabilities at longer wavelengths may at least partly explain the difference in attraction to FIL and LED lights. However, despite higher intensities above 665 nm for the FIL bulb relative to the LEDs (Figure 2), such small variances in intensity are unlikely to alter insect attraction significantly (Longcore et al., 2015). It is possible that the relatively large proportion of heat emitted as near infrared radiation (760–1400 nm) from the FIL bulb acted as a thermal attractant, especially to *Culicoides*, which have warm‐blooded hosts. A number of biting invertebrates use thermal gradients (Allan, Day, & Edman, 1987), and infrared detection has been recorded in others (Callahan, 1965, 1971; Evans, 1966). The thermal contrast between a warm light and the surrounding environment will vary with ambient temperature, for example, temperate versus tropical, and therefore, attractiveness may also vary. We recommend that future research controls for thermal emissions when investigating ecological consequences of artificial light at night. An alternative, but unlikely, explanation for greater attraction to the FIL light relative to the other lights is variation in electric fields; bumblebees (*Bombus terrestris*) can detect floral electric fields (Clarke, Whitney, Sutton, & Robert, 2013). The FIL light had a much higher power rating than the other lights we used, and it is feasible that the FIL light trap caught more insects than LEDs because of electrical rather than spectral or thermal differences, or a combination of these factors.

We found no difference in insect attraction between “cool‐” and “warm‐white” LEDs. Similar results were recorded in New Zealand (Pawson & Bader, 2014), whereas experiments on street lights in Germany showed “cool‐white” LEDs attracted more insects than “neutral/warm‐white” LEDs (Eisenbeis & Eick, 2011). These differences may be due to variations in experimental design and/or the habitats surveyed; these studies only sampled a single location, and there can be considerable variation between sites (Fig. S3). Furthermore, both studies and ours used slightly different lighting equipment and were conducted in different geographic locations where insect communities are likely to differ. Hence, there are too many variables to determine the underlying cause of these conflicting results. We used “off‐the‐shelf” lighting products to understand how current lighting may differ in attractiveness toward insects, whereas recent work using customized LEDs found that it is possible to reduce arthropod attraction to LED lights by making subtle changes to their spectral emissions (Longcore et al., 2015). Tuning LED spectral emissions may decrease insect attraction, although existing models do not accurately predict the attractiveness of non‐UV light sources such as LEDs to insects (Van Grunsven et al., 2014).

We found the attractiveness of the two LED lights differed between the two most abundant moth families we caught. More Crambidae (Pyraloidea) were caught around LEDW lights, whereas we found no difference in attraction for noctuid moths. Some noctuid moths possess a broad range of λmax values from UV to red (e.g., *Mamestra brassicae,* λmax = 360, 460, 540, 580 nm), whereas some pyralid moths show maximum absorption at longer wavelengths only (e.g., *Galleria mellonella,* λmax = 510 nm) (Briscoe & Chittka, 2001). These data support the difference in attraction between Crambidae and Noctuidae reported here, although more research is needed in this area. With the exception of a few well‐studied species (e.g., *Apis mellifera*), knowledge of insect spectral sensitivities remains poor, especially for nocturnal Lepidoptera.

The majority of insects attracted to the lights in our study were Diptera, of which approximately 15% were *Culicoides* biting midges (Ceratopogonidae). LEDs attracted fewer *Culicoides* than the other lights. Biting flies (including midges) can be diurnal, crepuscular, or nocturnal (Allan et al., 1987) and many are vectors of disease, especially in developing nations at lower latitudes (Jones et al., 2008). UV light attracts black flies (Simuliidae), mosquitoes (Culicidae), and tsetse flies (Glossinidae) (Allan et al., 1987), vectors of river blindness, malaria, and sleeping sickness, respectively. The use of non‐UV light sources, such as LEDs, in regions which harbor these and other insect vector species is therefore likely to be preferable to UV‐emitting lights, such as fluorescent (including CFL) and metal halide lights. Future studies should investigate the impact of LED lighting in the various climatic regions where these other vectors are likely to have human/animal health associations. “Blue‐green” light is also attractive to various biting flies (Bentley, Kaufman, Kline, & Hogsette, 2009; Bishop, Worrall, Spohr, McKenzie, & Barchia, 2004; Burkett & Butler, 2005), and the contrast between dark target objects and a brighter background is an important stimulus in host detection by species such as nocturnal mosquitoes and diurnal tsetse flies (Allan et al., 1987; Green & Cosens, 1983; Haufe, 1964). So broad‐spectrum “white” LEDs emitting “blue‐green” wavelengths and producing dark silhouettes of people and/or buildings may increase the number of interactions between biting flies and humans.

In comparison with developing nations at lower latitudes, countries in temperate regions have relatively few insect‐borne diseases of concern to human health. However, from a socioeconomic and animal welfare perspective, *Culicoides* spp. can transmit blue‐tongue virus and Schmallenberg virus, both causing serious illness in ruminants (Hoffmann et al., 2012; Maclachlan & Mayo 2013), African horse sickness virus (de Waal et al., 2016), as well as Oropouche virus to and between humans (Carpenter et al., 2013). Our finding that LEDs attract significantly fewer *Culicoides* than tungsten filament lights contradicts research by Bishop et al. (2004), who found that catching *Culicoides* spp. for national monitoring purposes was much more efficient with green LEDs than incandescent bulbs. However, Bishop et al. (2004) used incandescent lights with emissions largely confined to the visible light spectrum (400–700 nm), whereas infrared emissions from the FIL lights in this study may have contributed to the differences in our results. The possibility of thermal detection seems likely because red LEDs attracted fewer insects than incandescent lights for all eight of the *Culicoides* species they tested (Bishop et al., 2004).

Our insect traps caught insects that flew very close to the light itself. Not all insects behave in this way, and therefore, our results are skewed towards taxa that do. An insect vector that flies directly towards a light source may pose a lower threat to susceptible hosts than one which is attracted to an illuminated area around the light where humans and/or livestock are more likely to be found. Use of multiple insect trapping devices may have caught a wider variety of vector taxa.

Our finding that domestic LEDs attract fewer insects than other lighting technologies is consistent with most (Eisenbeis & Eick, 2011; Poiani et al., 2015; Van Grunsven et al., 2014) but not all (Bishop et al., 2004; Pawson & Bader, 2014) previous studies. Further work is needed to understand these differences. In particular, we encourage future research to include experimental studies conducted in heterogeneous urban environments. Our results indicate that the use of LEDs instead of CFL and tungsten filament lights should result in fewer disturbances to wildlife, have the potential to mitigate existing human–wildlife conflicts arising from light pollution, help reduce the health risk to humans and livestock, and limit socioeconomic impacts. Thus, choice of light has human health and animal welfare implications which have largely been overlooked in similar studies investigating the impact of artificial light on wildlife and ecosystems (although see Barghini and de Medeiros (2010)).

However, exterior LEDs should not be considered a lighting panacea. Ongoing improvements to LED technology (e.g., Kong, Ibbetson, & Edmond, 2014) are decreasing product costs and are likely to result in the installation of LEDs in previously unlit areas. If the installation of cheaper LED lighting encourages people to stay active for longer, this will enhance the potential for those insects attracted to lights to affect human health (Barghini & de Medeiros, 2010).

Furthermore, many insect vector species worldwide are diurnal (Allan et al., 1987), and the introduction of permanent lighting fixtures may lead to changes in insect behavior such that diurnal vector species become nocturnally active. Temporal changes in behavior include birds singing around street lights (Byrkjedal, Lislevand, & Vogler, 2012; Da Silva, Valcu, & Kempenaers, 2015), alteration in shorebird foraging behavior (Dwyer, Bearhop, Campbell, & Bryant, 2013; Santos et al., 2010), and diurnal spiders stalking insect prey around lights at night (Frank, 2009). Similar changes in insect behavior may have human health, social, economic, animal welfare, and ecological implications.

## Conflict of Interest

None declared.

## Data Accessibility

Relevant supporting data are available from the Dryad Digital Repository: http://dx.doi.org/10.5061/dryad.pn4m4.
