# Supplementary material for: Experimentally comparing the attractiveness of domestic lights to insects: Do LEDs attract fewer insects than conventional light types?
Source: Ecol Evol. 2016 Oct 13;6(22):8028–36. doi: 10.1002/ece3.2527 (PMC5108255; doi:10.1002/ece3.2527)

**Fig. S1.** Schematic illustrating the light trap design used in this study. The box on the ground contained one or two battery packs and a current inverter. A collection beaker was attached to a funnel which was suspended below the light bulb using fishing wire such that the top of the funnel was horizontally in line with the bottom of the bulb. The lamp was attached to a horizontal piece of metal doweling at a height of 1.3 m and was positioned 20 cm from the vertical doweling.


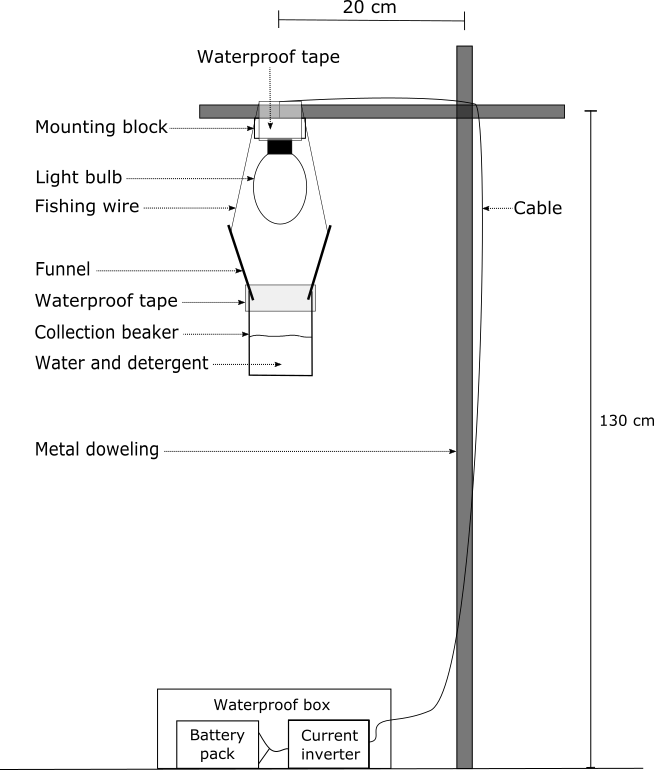

Supplement: Supplementary file 1 [file ECE3-6-8028-s001.docx]
