# Supplementary material for: Experimentally comparing the attractiveness of domestic lights to insects: Do LEDs attract fewer insects than conventional light types?
Source: Ecol Evol. 2016 Oct 13;6(22):8028–36. doi: 10.1002/ece3.2527 (PMC5108255; doi:10.1002/ece3.2527)

**Fig. S2.** Pooled counts of Diptera, grouped by family, caught at all four light traps during the ‘evening’ sampling periods (n = 17 sites).


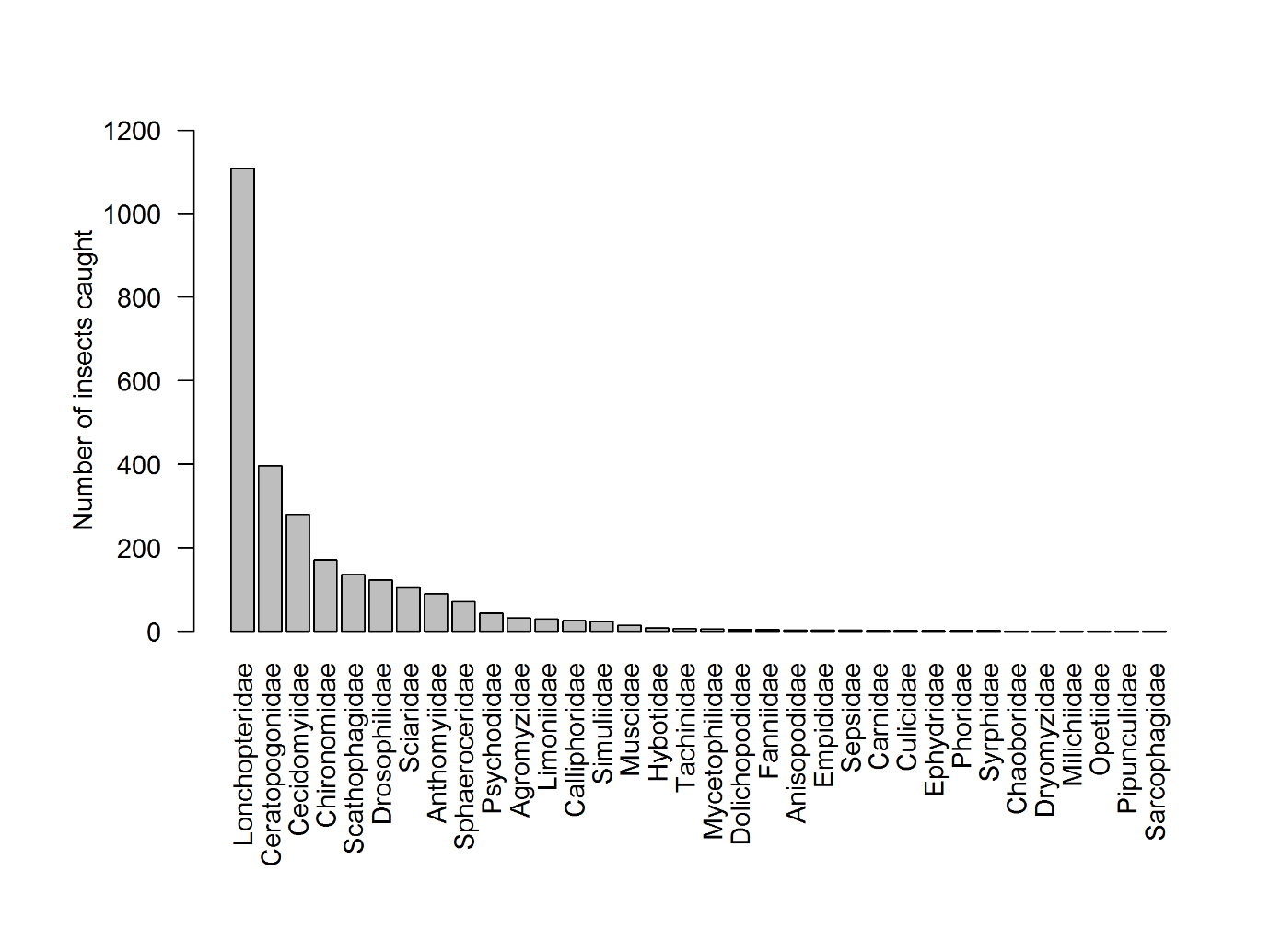

Supplement: Supplementary file 2 [file ECE3-6-8028-s002.docx]
