# Supplementary material for: Experimentally comparing the attractiveness of domestic lights to insects: Do LEDs attract fewer insects than conventional light types?
Source: Ecol Evol. 2016 Oct 13;6(22):8028–36. doi: 10.1002/ece3.2527 (PMC5108255; doi:10.1002/ece3.2527)

**Fig. S3.** Mosaic plot of ‘evening’ insect catch by site (n = 17) and per light (n = 4). Sites are denoted by letter; CFL denotes compact fluorescent, FIL tungsten filament, LEDC light-emitting diode ‘cool-white’, and LEDW light-emitting diode ‘warm-white’ lights. Column widths are proportional to the size of insect catch over four-hour sampling periods commencing at sunset.


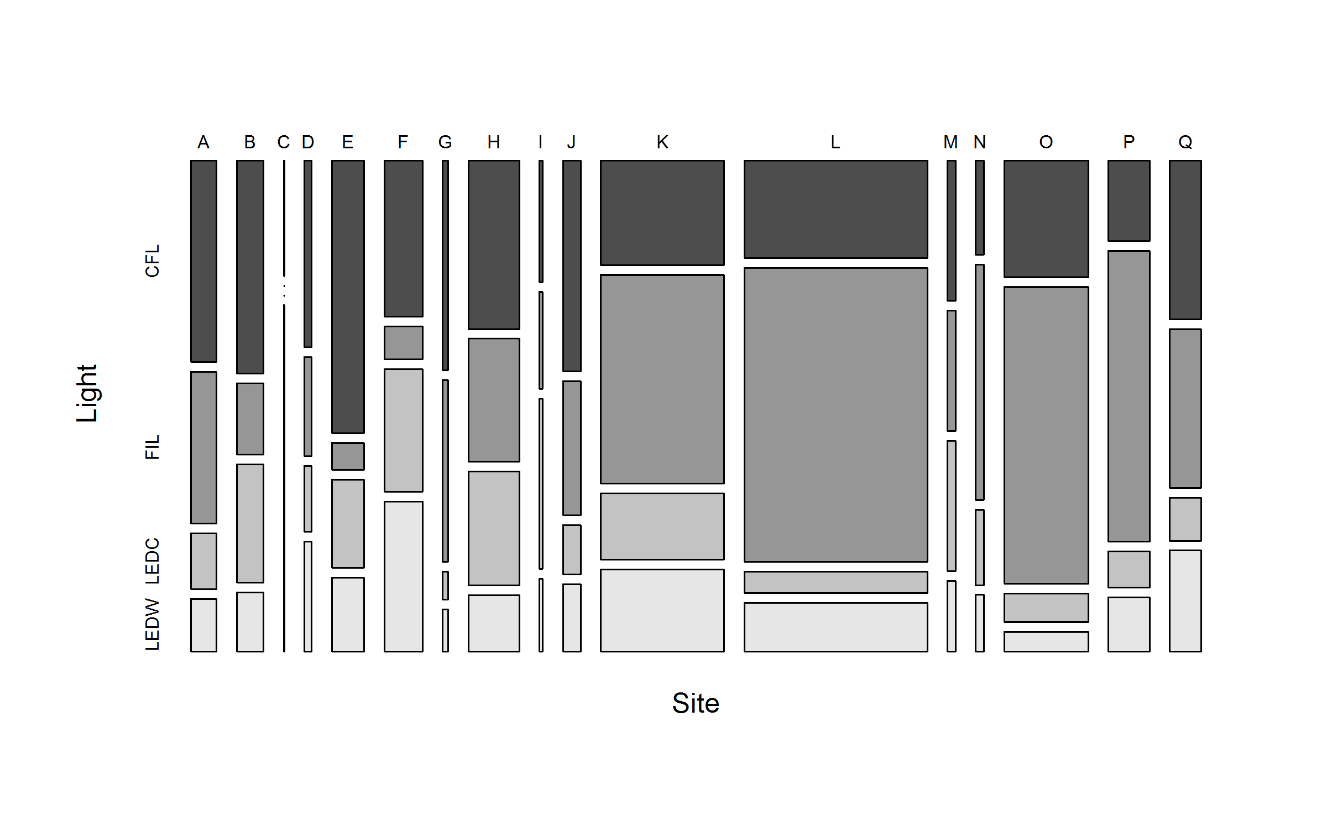

Supplement: Supplementary file 3 [file ECE3-6-8028-s003.docx]
