# Supplementary material for: Experimentally comparing the attractiveness of domestic lights to insects: Do LEDs attract fewer insects than conventional light types?
Source: Ecol Evol. 2016 Oct 13;6(22):8028–36. doi: 10.1002/ece3.2527 (PMC5108255; doi:10.1002/ece3.2527)
Supplement: Supplementary file 4 [file ECE3-6-8028-s004.docx]

**Table S1.** Total number of insects caught by family during ‘evening’ trapping sessions at the four lights used in this experiment. Lights were compact fluorescent (CFL), filament (FIL), ‘cool-white’ light-emitting diode (LEDC) and ‘warm-white’ light-emitting diode (LEDW).

| Order | Family | CFL | FIL | LEDC | LEDW |
| --- | --- | --- | --- | --- | --- |
| Coleoptera | Byrrhidae |  | 1 |  |  |
|  | Cantharidae | 3 | 9 |  |  |
|  | Chrysomelidae |  | 1 |  |  |
|  | Cryptophagidae | 1 | 1 | 1 |  |
|  | Hydrophilidae | 4 | 10 | 2 | 1 |
|  | Nitidulidae |  |  | 2 |  |
|  | Scarabaeidae | 8 | 3 |  |  |
|  | Staphylinidae | 1 |  |  | 1 |
|  | Tenebrionidae |  |  | 1 | 1 |
| Dermaptera | Forficulidae | 2 |  |  |  |
| Diptera | Agromyzidae | 6 | 15 | 5 | 6 |
|  | Anisopodidae | 3 |  |  |  |
|  | Anthomyiidae | 21 | 17 | 40 | 13 |
|  | Calliphoridae | 10 | 4 | 8 | 4 |
|  | Carnidae | 1 |  | 1 |  |
|  | Cecidomyiidae | 12 | 236 | 4 | 29 |
|  | Ceratopogonidae | 57 | 320 | 7 | 13 |
|  | Chaoboridae | 1 |  |  |  |
|  | Chironomidae | 36 | 77 | 21 | 37 |
|  | Culicidae | 1 | 1 |  |  |
|  | Dolichopodidae |  | 4 |  | 1 |
|  | Drosophilidae | 32 | 68 | 15 | 9 |
|  | Dryomyzidae |  |  |  | 1 |
|  | Empididae |  |  | 2 | 1 |
|  | Ephydridae |  | 1 |  | 1 |
|  | Fanniidae |  |  |  | 4 |
|  | Hybotidae | 3 | 5 |  |  |
|  | Limoniidae | 15 | 11 | 4 |  |
|  | Lonchopteridae | 253 | 644 | 79 | 132 |
|  | Milichiidae |  |  | 1 |  |
|  | Muscidae | 2 | 2 | 7 | 3 |
|  | Mycetophilidae | 1 | 2 | 1 | 2 |
|  | Opetiidae |  | 1 |  |  |
|  | Phoridae | 1 |  |  | 1 |
|  | Pipunculidae |  | 1 |  |  |
|  | Psychodidae | 2 | 32 |  | 10 |
|  | Sarcophagidae | 1 |  |  |  |
|  | Scathophagidae | 34 | 17 | 43 | 42 |
|  | Sciaridae | 32 | 51 | 10 | 12 |
|  | Sepsidae |  | 1 |  | 2 |
|  | Simuliidae | 10 | 4 | 7 | 3 |
|  | Sphaeroceridae | 20 | 22 | 17 | 12 |
|  | Syrphidae |  | 2 |  |  |
|  | Tachinidae | 2 |  | 2 | 3 |
| Ephemeroptera | Baetidae |  | 1 |  |  |
|  | Caenidae |  | 1 |  |  |
| Hemiptera | Aphididae | 2 |  | 2 |  |
|  | Aphidoidea | 5 | 9 | 5 | 2 |
|  | Cicadellidae |  | 2 |  |  |
|  | Corixidae |  | 1 |  |  |
|  | Delphacidae | 1 |  |  |  |
|  | Miridae | 13 | 3 | 5 | 5 |
| Hymenoptera | Braconidae | 9 | 13 | 4 | 5 |
|  | Cynipidae |  | 1 |  |  |
|  | Eulophidae | 2 |  |  |  |
|  | Figitidae | 1 |  |  | 1 |
|  | Formicidae | 2 | 2 |  | 4 |
|  | Ichneumonidae | 3 | 8 |  | 3 |
|  | Platygastridae |  | 3 |  |  |
|  | Proctotrupidae | 1 |  |  |  |
|  | Pteromalidae | 1 | 1 | 1 |  |
| Lepidoptera | Arctiidae | 11 | 5 | 1 |  |
|  | Blastobasidae | 1 | 2 |  | 2 |
|  | Coleophoridae |  |  |  | 1 |
|  | Crambidae | 86 | 74 | 12 | 34 |
|  | Drepanidae | 1 |  |  |  |
|  | Gelechiidae |  | 1 |  | 1 |
|  | Geometridae | 6 | 9 |  | 1 |
|  | Hepialidae | 1 |  |  |  |
|  | Lasiocampidae |  | 3 |  |  |
|  | Noctuidae | 72 | 70 | 13 | 9 |
|  | Tortricidae | 9 | 4 | 1 | 1 |
|  | Unidentified | 7 | 11 | 1 | 2 |
| Neuroptera | Chrysopidae |  | 2 |  |  |
| Psocoptera | Ectopsocidae |  | 1 |  | 1 |
|  | Psocidae |  |  | 1 |  |
| Trichoptera | Hydropsychidae | 2 | 1 |  |  |
|  | Hydroptilidae | 1 |  |  |  |
|  | Leptoceridae |  | 1 |  |  |
|  | Polycentropodidae |  |  |  | 1 |
|  | Unidentified |  | 3 |  |  |
