# Supplementary material for: Experimentally comparing the attractiveness of domestic lights to insects: Do LEDs attract fewer insects than conventional light types?
Source: Ecol Evol. 2016 Oct 13;6(22):8028–36. doi: 10.1002/ece3.2527 (PMC5108255; doi:10.1002/ece3.2527)
Supplement: Supplementary file 5 [file ECE3-6-8028-s005.docx]

**Table S2.** Results of multiple comparison tests applied to GLMMs for ‘evening’ insect catches (n = 16 sites): (a) total insects; (b) Diptera; and (c) Lepidoptera. The number of insects caught at one site (“L”) was unusually large, especially for the FIL lamp, and so was excluded from this analysis. In all models ‘light position’ nested within ‘site’ was included as a random effect and light as the only fixed effect term. Lights were compact fluorescent (CFL), filament (FIL), ‘cool-white’ light-emitting diode (LEDC) and ‘warm-white’ light-emitting diode (LEDW). * indicates a significant (p <0.05) difference

| a) |  | Estimate | SE | Z value | P |
| --- | --- | --- | --- | --- | --- |
|  | FIL – CFL | 0.425 | 0.196 | 2.17 | 0.132 |
|  | LEDC – CFL | -0.721 | 0.207 | -3.49 | 0.003 * |
|  | LEDW – CFL | -0.657 | 0.206 | -3.19 | 0.008 * |
|  | LEDC – FIL | -1.146 | 0.204 | -5.61 | <0.001 * |
|  | LEDW – FIL | -1.082 | 0.204 | -5.32 | <0.001 * |
|  | LEDW ̶ LEDC | 0.064 | 0.213 | 0.23 | 0.991 |
| b) |  | Estimate | SE | Z value | P |
|  | FIL – CFL | 0.578 | 0.244 | 2.37 | 0.084 |
|  | LEDC – CFL | -0.360 | 0.243 | -1.42 | 0.485 |
|  | LEDW – CFL | -0.346 | 0.253 | -1.37 | 0.521 |
|  | LEDC – FIL | -0.938 | 0.249 | -3.77 | 0.001 * |
|  | LEDW – FIL | -0.924 | 0.249 | -3.71 | 0.001 * |
|  | LEDW ̶ LEDC | 0.014 | 0.257 | 0.05 | 0.999 |
| c) |  | Estimate | SE | Z value | P |
|  | FIL – CFL | -0.021 | 0.151 | -0.14 | 0.999 |
|  | LEDC – CFL | -1.857 | 0.233 | -7.98 | <0.001 * |
|  | LEDW – CFL | -1.274 | 0.193 | -6.61 | <0.001 * |
|  | LEDC ̶ FIL | -1.836 | 0.232 | -7.91 | <0.001 * |
|  | LEDW – FIL | -1.253 | 0.195 | -6.43 | <0.001 * |
|  | LEDW ̶ LEDC | 0.583 | 0.262 | 2.22 | 0.113 |
