# Supplementary material for: Experimentally comparing the attractiveness of domestic lights to insects: Do LEDs attract fewer insects than conventional light types?
Source: Ecol Evol. 2016 Oct 13;6(22):8028–36. doi: 10.1002/ece3.2527 (PMC5108255; doi:10.1002/ece3.2527)
Supplement: Supplementary file 6 [file ECE3-6-8028-s006.docx]

**Table S3.** Results of a multiple comparison test applied to a negative binomial GLMM for ‘all night’ total insect catches (n = 11 sites). ‘Light position’ nested within ‘site’ were included as random effects and ‘light’ as the only fixed effect term. Lights were compact fluorescent (CFL), filament (FIL), ‘cool-white’ light-emitting diode (LEDC) and ‘warm-white’ light-emitting diode (LEDW). * indicates a significant (p <0.05) difference

|  | Estimate | SE | Z value | P |
| --- | --- | --- | --- | --- |
| LEDC – CFL | -0.770 | 0.205 | -3.759 | <0.001 * |
| LEDW – CFL | -0.615 | 0.204 | -3.011 | 0.007 * |
| LEDW ̶ LEDC | 0.155 | 0.213 | 0.731 | 0.745 |
